# The PDK1 Inhibitor Dichloroacetate Controls Cholesterol Homeostasis Through the ERK5/MEF2 Pathway

**DOI:** 10.1038/s41598-017-10339-5

**Published:** 2017-09-06

**Authors:** Abrar Ul Haq Khan, Nerea Allende-Vega, Delphine Gitenay, Sabine Gerbal-Chaloin, Claire Gondeau, Dang-Nghiem Vo, Sana Belkahla, Stefania Orecchioni, Giovanna Talarico, Francesco Bertolini, Milica Bozic, Jose M. Valdivielso, Fabienne Bejjani, Isabelle Jariel, Isabel C. Lopez-Mejia, Lluis Fajas, Charles-Henri Lecellier, Javier Hernandez, Martine Daujat, Martin Villalba

**Affiliations:** 1 INSERM, U1183; Université de Montpellier, UFR Medecine, 80, av. Augustin Fliche, 34295 Montpellier Cedex 5, France; 2 Institut de Médecine Régénératrice et Biothérapie (IRMB), CHU Montpellier, Montpellier, 34295 France; 3 Département d’Hépato-gastroentérologie A, Hôpital Saint Eloi, CHU, Montpellier, France; 4 Laboratory of Hematology-Oncology, European Institute of Oncology, Milan, Italy; 5 Vascular and Renal Translational Research Group. Institut de Recerca Biomedica de Lleida (IRBLLIDA), Lleida, Spain; 6 IGMM, CNRS, Univ. Montpellier, Montpellier, France; 7 Center for Integrative Genomics, University of Lausanne, Lausanne, Switzerland

## Abstract

Controlling cholesterol levels is a major challenge in human health, since hypercholesterolemia can lead to serious cardiovascular disease. Drugs that target carbohydrate metabolism can also modify lipid metabolism and hence cholesterol plasma levels. In this sense, dichloroacetate (DCA), a pyruvate dehydrogenase kinase (PDK) inhibitor, augments usage of the glycolysis-produced pyruvate in the mitochondria increasing oxidative phosphorylation (OXPHOS). In several animal models, DCA decreases plasma cholesterol and triglycerides. Thus, DCA was used in the 70 s to treat diabetes mellitus, hyperlipoproteinemia and hypercholesterolemia with satisfactory results. However, the mechanism of action remained unknown and we describe it here. DCA increases *LDLR* mRNA and protein levels as well as LDL intake in several cell lines, primary human hepatocytes and two different mouse models. This effect is mediated by transcriptional activation as evidenced by H3 acetylation on lysine 27 on the *LDLR* promoter. DCA induces expression of the MAPK ERK5 that turns on the transcription factor MEF2. Inhibition of this ERK5/MEF2 pathway by genetic or pharmacological means decreases LDLR expression and LDL intake. In summary, our results indicate that DCA, by inducing OXPHOS, promotes ERK5/MEF2 activation leading to LDLR expression. The ERK5/MEF2 pathway offers an interesting pharmacological target for drug development.

## Introduction

Elevated level of low-density lipoprotein (LDL) in blood is a predominant risk factor for atherosclerosis, a large cause of mortality^1^. Control of plasma cholesterol levels largely depends on low-density lipoprotein receptor (LDLR), which mediates the endocytosis of cholesterol-rich LDL^2, 3^. This process takes place mainly in the liver^2, 3^. LDL is degraded in lysosomes and cholesterol made available for repression of microsomal enzyme 3-hydroxy-3-methylglutaryl coenzyme A (HMG CoA) reductase, the rate-limiting step in cholesterol synthesis^2, 3^. Hence, LDLR regulates intracellular and extracellular cholesterol homeostasis and is involved in atherosclerosis due to accumulation of LDL-cholesterol in blood^4^.

Lipid and carbohydrate metabolic pathways are interconnected and targeting the latter may result in altered cholesterol levels. The pyruvate dehydrogenase (PDH) kinase (PDK) inhibitor dichloroacetate (DCA) activates PDH, the rate-limiting enzyme of aerobic glucose oxidation^5^. PDH converts glycolysis-produced pyruvate in acetyl-CoA that enters the mitochondria and is consumed in the process of oxidative phosphorylation (OXPHOS). Thus, DCA inhibits glycolysis and lactate production and induces OXPHOS^6–10^. DCA concentration in DCA-treated patients is unclear because its half-life is less than 1 hour and it is not detectable in patients during the initial phase of treatment that can last 2 to 3 months^11, 12^. However, DCA inhibits its own metabolism and serum concentrations increase, eventually reaching a plateau, with plasma concentrations around 0.3 mM^11^. Michelakis *et al*. gave 50 mg/Kg/day of DCA to patients and obtained similar values: 0.44 ± 0.16 mM^12^.

DCA treatment decreases plasma triglyceride and cholesterol in animal models and humans^13, 14^. This effect is likely indirect due to the decrease in plasma of very-low-density lipoproteins (VLDL) by stimulation of triglyceride oxidation^5, 14^, although the mechanism remains unknown. After these findings there was an attempt to use DCA to treat two cases of familial hypercholesterolemia (FH)^15^. DCA reduced circulating cholesterol levels in both patients by decreasing LDL cholesterol^15^. Unfortunately, DCA had to be discontinued following neuropathological effects and this precluded its use to treat high cholesterol levels^15^. The biological mechanism underlying DCA effects on cholesterol was unknown and, to the best of our knowledge, no further studies were conducted to investigate it. Nowadays, the most accepted explanation would be that the change in carbohydrate metabolism alters lipid metabolism. In this sense, DCA, by inhibiting glycolysis, activates AMPK^9^, which inhibits cholesterol synthesis^16, 17^ leading to LDLR expression^18, 19^. Enhanced LDLR expression is mediated by a MAPKK because the MAPKK inhibitors PD98059^18^ and U0126^20, 21^ restrain it. This would indicate that DCA has a similar mechanism of action than the alkaloid berberine and its analogs^18, 19^. An alternative explanation could be that DCA induces ROS production^9, 10^ and the cellular oxidative state regulates LDLR expression^22^. Hence, changes in ROS could mediate DCA-induced LDLR expression.

In preliminary experiments, we found by transcriptome analysis that *LDLR* was one of the most downregulated genes in hematopoietic cells expressing a small hairpin RNA for ERK5 (shERK5) and one of the most upregulated after DCA treatment. We have previously observed that DCA increased mRNA and protein expression of the MAPK ERK5, which is essential for cell survival in OXPHOS conditions^8, 10, 23–26^. ERK5 activates the MEF2 family of transcription factors^27–30^ that in turn mediates most of the effects of ERK5 on metabolism^8, 10, 23–26^. In fact, DCA activates multiple genes with promoters containing MEF2 binding sites. Genomic analysis using the UCSC genome browser (http://genome.ucsc.edu/) showed that *LDLR* is one of such genes because its promoter contains several binding sites for at least two MEF2 proteins, MEF2A and MEF2C, which have been validated in several cell lines by Chromatin Immunoprecipitation (ChIP; (http://genome.ucsc.edu/). We investigate here how DCA reduces cholesterol levels and the role of the ERK5/MEF2 pathway.

## Results

### DCA enhances LDLR expression

**Figure 1:**
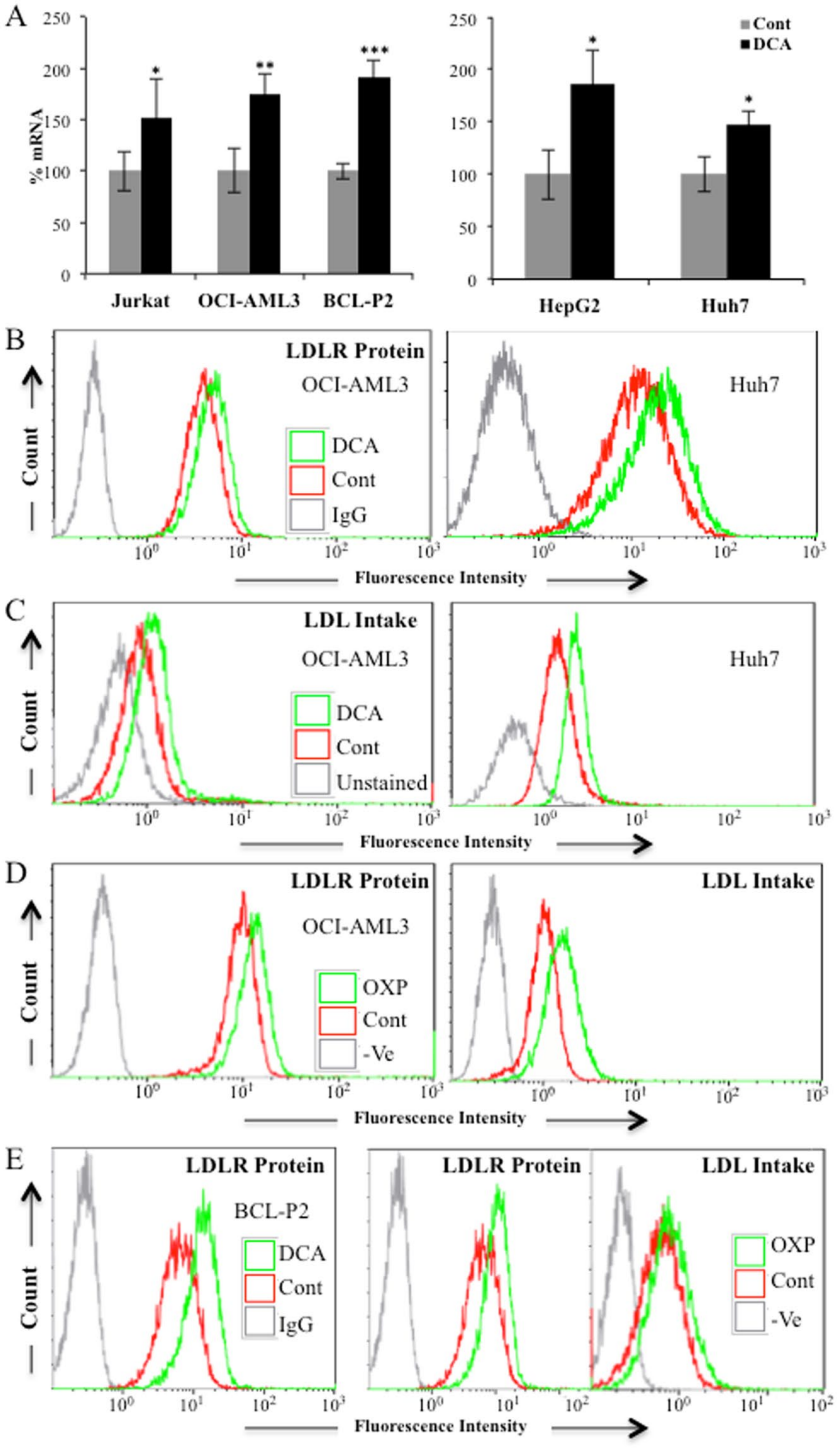
OXPHOS induced LDLR expression and LDL uptake. (**A**) The hematopoietic cell lines Jurkat and OCI-AML3 and primary cells from a BCL patient (BCL-P2) as well as HepG2-C3A and Huh7 hepatic cell lines were treated with 10 mM DCA for 24 h and *LDLR* mRNA was analyzed by RT-qPCR. (**B**) Cell lines were treated for 72 h with 5 mM DCA and LDLR protein in plasma membrane was analyzed by FACs. (**C**) Cells were treated as in (**B**) and fluorescent LDL intake analyzed by FACs. (**D**) OCI-AML3 cells were grown in OXPHOS medium for 2 weeks and LDLR expression (left) and LDL intake (right) were analyzed by FACs. (**E**) BCL-P2 cells were treated with 5 mM DCA for 1 week (left) or were grown in OXPHOS medium for 2 weeks (center) and LDLR protein in plasma membrane analyzed by FACs. LDL intake (right) was analyzed in cells growing in OXPHOS. The bar graphs represent means ± SD of 3 independent experiments performed in triplicate; *p < 0.05, **p < 0.01, ***p < 0.005 student t-test compared to control cells.

We first observed that DCA increased *LDLR* mRNA in hematopoietic cells (Fig. 1A, left panel). Since liver is the main organ that takes up LDL-cholesterol from blood, we tested the effect of DCA in two hepatic cell lines, finding that *LDLR* mRNA levels were also increased (Fig. 1A, right panel). Augmented *LDLR* mRNA levels correlated with an increase of LDLR protein in the plasma membrane (Fig. 1B and Supplemental Fig. 1A). We then tested the functional consequence of this enhanced expression by incubating control or DCA-treated cells with fluorescently labeled LDL. DCA increased LDL transport into these cell lines (Fig. 1C and Supplemental Fig. 1B).

### Cells performing OXPHOS increase LDLR activity

DCA induces OXPHOS in leukemic cells^8–10, 24, 25, 31^. To investigate whether the metabolic switch from aerobic glycolysis to OXPHOS mediated by DCA affect LDLR expression, we used a glucose-free culture medium containing a final glutamine concentration of 4 mM and 10 mM galactose. Glutamine is used to drive mitochondria to utilize OXPHOS and galactose allows cells to synthesize nucleic acids through the pentose phosphate pathway^8, 24, 32, 33^. We called it ‘OXPHOS medium’ because it forced leukemic cells to use OXPHOS as primary ATP source^8, 9, 23^. We observed that acute myeloid leukemia (AML) OCI-AML3 cells growing in OXPHOS medium for 2 weeks, such as those treated with DCA, presented an increase of *ERK5* and *LDLR* mRNA (Supplemental Fig. 2A), LDLR protein and LDL intake (Fig. 1D). These results indicated that, as expected, the effect of DCA on LDLR expression was due to a metabolic switch. DCA and OXPHOS also increased *LDLR* mRNA and protein as well as LDL intake in primary lymphoma cells derived from a B cell lymphoma patient (BCL-P2; Fig. 1A, Supplemental Fig. 1B and Fig. 1E). We found similar results in the hepatic cell lines HepG2-C3A and HuH7, with OXPHOS media increasing LDLR protein and uptake (Supplemental Fig. 3).

**Figure 2:**
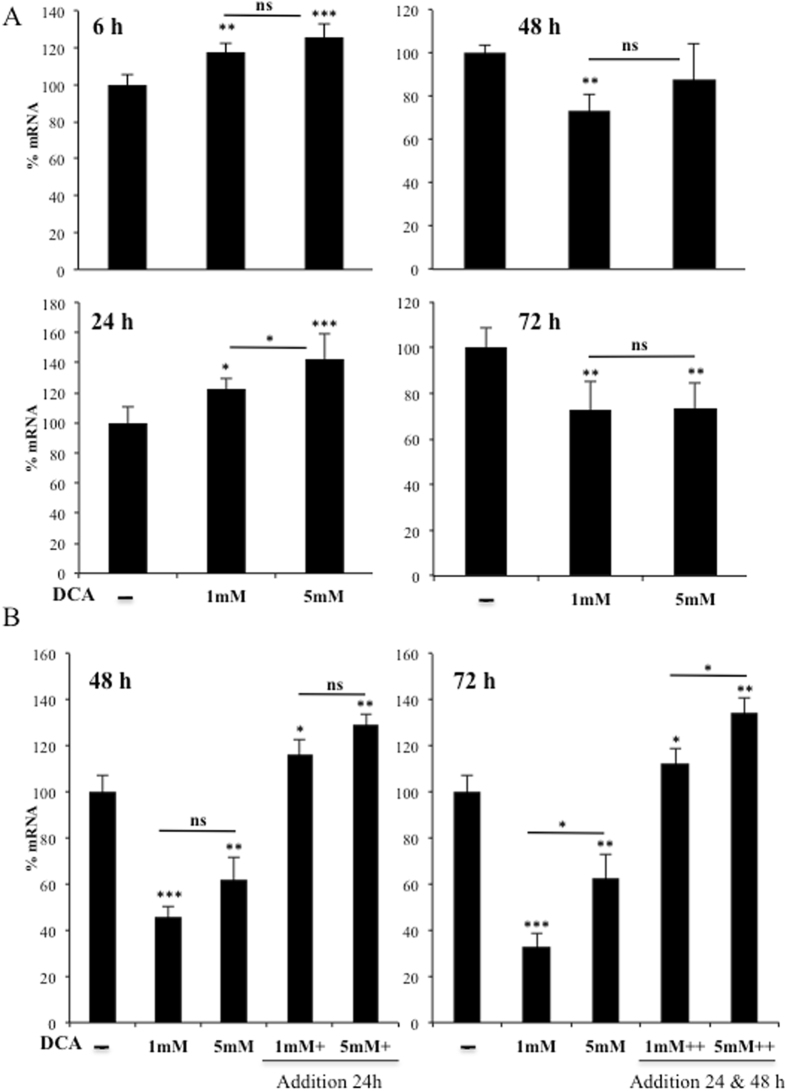
DCA induced LDLR expression in primary human hepatocytes. (**A**) Primary hepatocytes were treated with the indicated concentrations of DCA for the indicated times. (**B**) Cells were treated at time 0 with DCA and some were treated every 24 h before harvesting as indicated. *LDLR* mRNA was analyzed by RT-qPCR. The bar graphs represent means ± SD of 3 independent donors performed in triplicate; *p < 0.05, **p < 0.01, ***p < 0.005 one-way ANOVA with post-hoc Tukey test.

In primary human hepatocytes, DCA also induced LDLR expression at 6 and 24 h post treatment (Fig. 2A). However, effects disappeared at 48 and 72 h with a net decrease (Fig. 2A). This is likely due to the short DCA half-life, since *LDLR* mRNA levels were kept high when fresh DCA was added to the medium every 24 h (Fig. 2B).

### Reactive Oxygen Species (ROS) do not mediate DCA-induced LDLR expression

**Figure 3:**
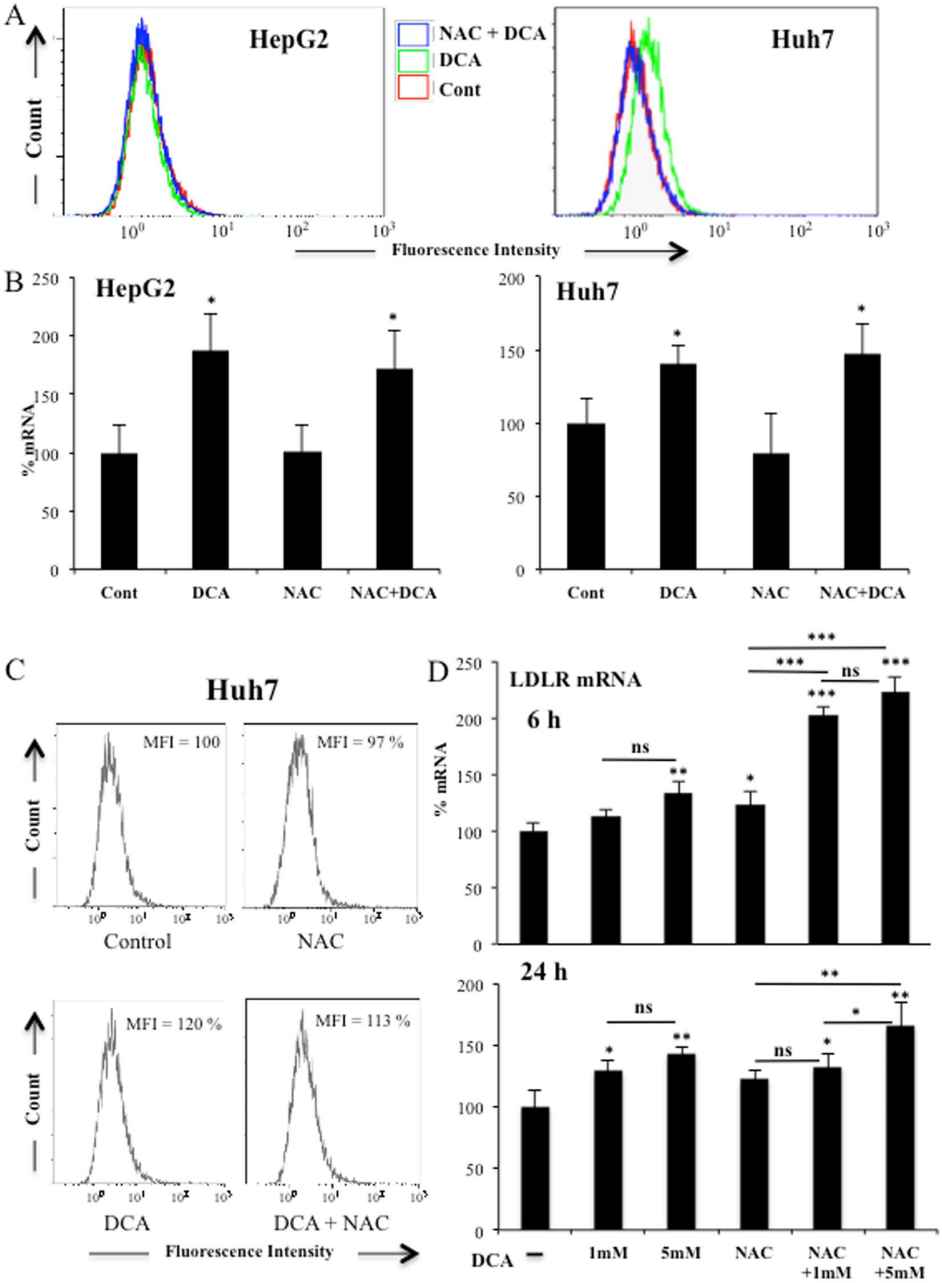
Increase in ROS levels was not required for LDLR expression. (**A**) Both hepatic cell lines were treated with 2 mM NAC 1 h before adding DCA (10 mM) for 24 h. Cells were labeled with CH-H2DCFDA and analyzed by FACs for ROS production. *LDLR* mRNA (**B**) or protein (**C**) from these cells were analyzed as described in Fig. 1. Results represent the means ± SD of these donors with experiments performed in triplicate. The data represent means ± SD; *p < 0.05, **p < 0.01, ***p < 0.005 student t-test compared to cells non treated with DCA. D) Primary hepatocytes from 2 independent donors were treated for 6 and 24 h as in (**A**) but with two different DCA concentrations before analyzing *LDLR* mRNA expression. The data represent means ± SD; *p < 0.05, **p < 0.01, ***p < 0.005 one-way ANOVA with post-hoc Tukey test.

The cellular oxidative state can regulate LDLR expression^22^ and DCA induces ROS production in certain hematopoietic cell lines but not in others^9, 10^. We observed a similar phenomenon in two different hepatic cell lines. In Huh7 cells there was an increase of ROS, but not in HepG2-C3A cells, after DCA treatment (Fig. 3A). In contrast, in all cell lines utilized in this study, DCA increased LDLR expression, suggesting that ROS production was not essential (Figs 1A and 3B). Next, we incubated both hepatic cell lines with the antioxidant N-acetyl-cysteine (NAC). NAC efficiently blocked DCA-induced ROS production in HuH7 cells (Fig. 3A); however, it did not affect DCA-induced *LDLR* mRNA (Fig. 3B) or plasma membrane protein (Fig. 3C). To definitively exclude that ROS played any role in LDLR induction, we incubated primary hepatocytes with DCA in presence or absence of NAC. We found that NAC did not inhibit and in fact increased *LDLR* mRNA expression (Fig. 3D). These results exclude a major role for *de novo* ROS production in LDLR expression after DCA treatment.

### DCA induces LDLR *in vivo*

**Figure 4:**
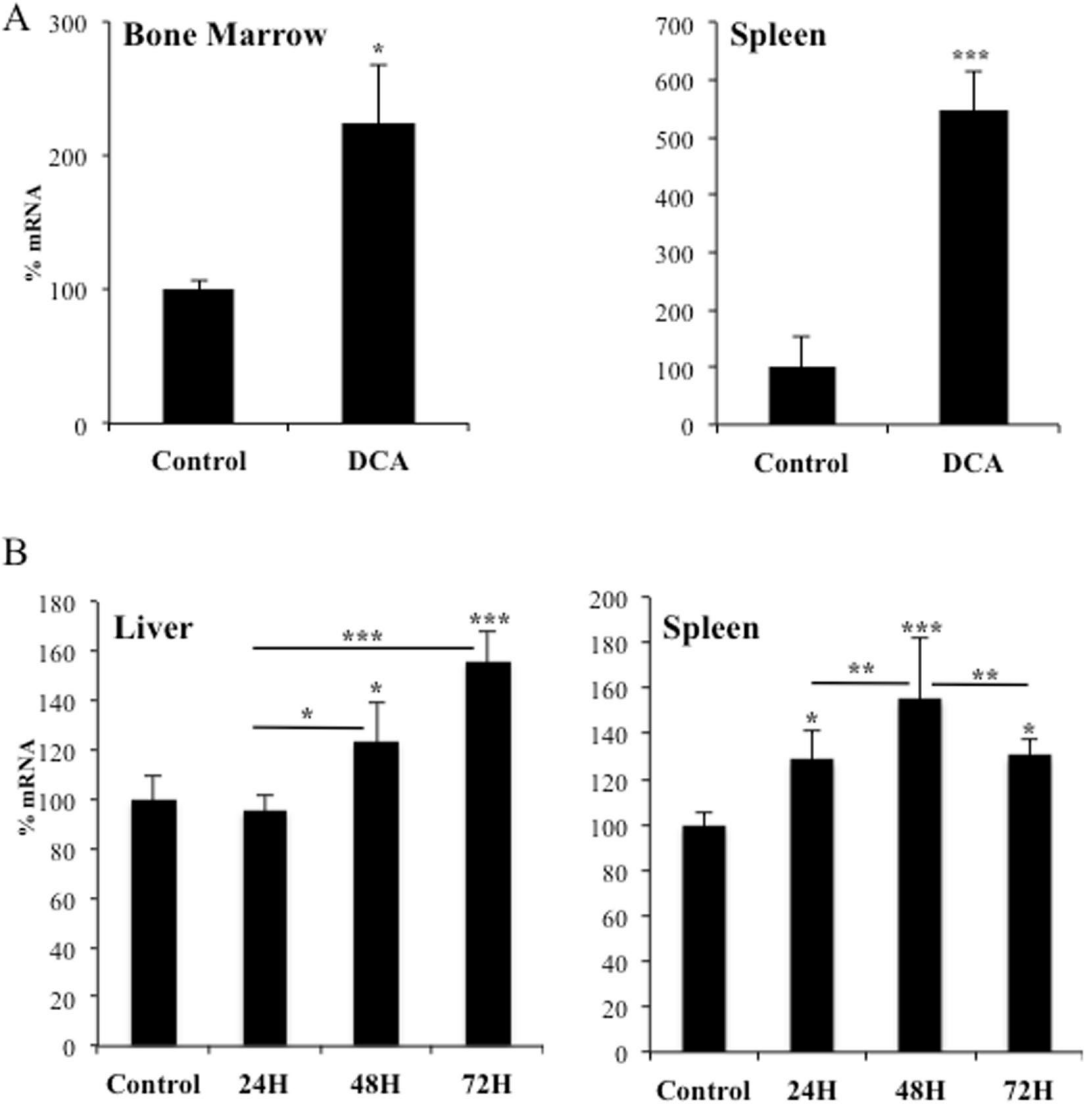
DCA induced LDLR expression *in vivo*. (**A**) NSG mice were engrafted with primary human AML cells. At day 80 post-graft, they were treated with DCA (n = 4) or leave untreated (n = 4). At day 140, mRNA from bone marrow or spleen was isolated and human *LDLR* mRNA expression was quantified by qPCR. The data represent means ± SD; *p < 0.05, **p < 0.01, ***p < 0.005 student t-test compared to non treated mice. (**B**) B6 wt mice (n = 4/5 per group) were treated with a dose of DCA (50 mg/kg) everyday intraperitoneally and mouse *LDLR* mRNA was analyzed in spleen and liver at indicated time points. The data represent means ± SD; *p < 0.05, **p < 0.01, ***p < 0.005 one-way ANOVA with post-hoc Tukey test.

We next assessed whether DCA enhances *LDLR* expression *in vivo*. We engrafted human AML primary cells in non-obese diabetic/severe combined immunodeficient (NOD/SCID)-interleukin-2 receptor γ chain null (NSG) mice, as previously described^9, 10^. Mice with established tumors (day 80 post-graft) were treated daily with DCA (Fig. 4A). The treatment was not toxic and did not show any notable effect on mice survival^9^. Human tumor AML cells gather in murine spleen and bone marrow, hence we isolated mRNA from these organs. We used human-specific primers and observed that DCA significantly increased expression of *LDLR* mRNA (Fig. 4A).

We also found augmented mouse *LDLR* mRNA levels in normal liver and spleen from wt mice that were treated daily, for 1 and up to 3 days, with DCA (Fig. 4B). The effect was first observed in hematopoietic cells gathering in spleen and, later, in liver. Thus, DCA induced LDLR expression in multiple cell populations *in vivo*. This could, at least partially, explain the reduction in plasma cholesterol levels after DCA treatment in several species including humans^5, 13–15, 34^.

### ERK5 regulates LDLR expression

**Figure 5:**
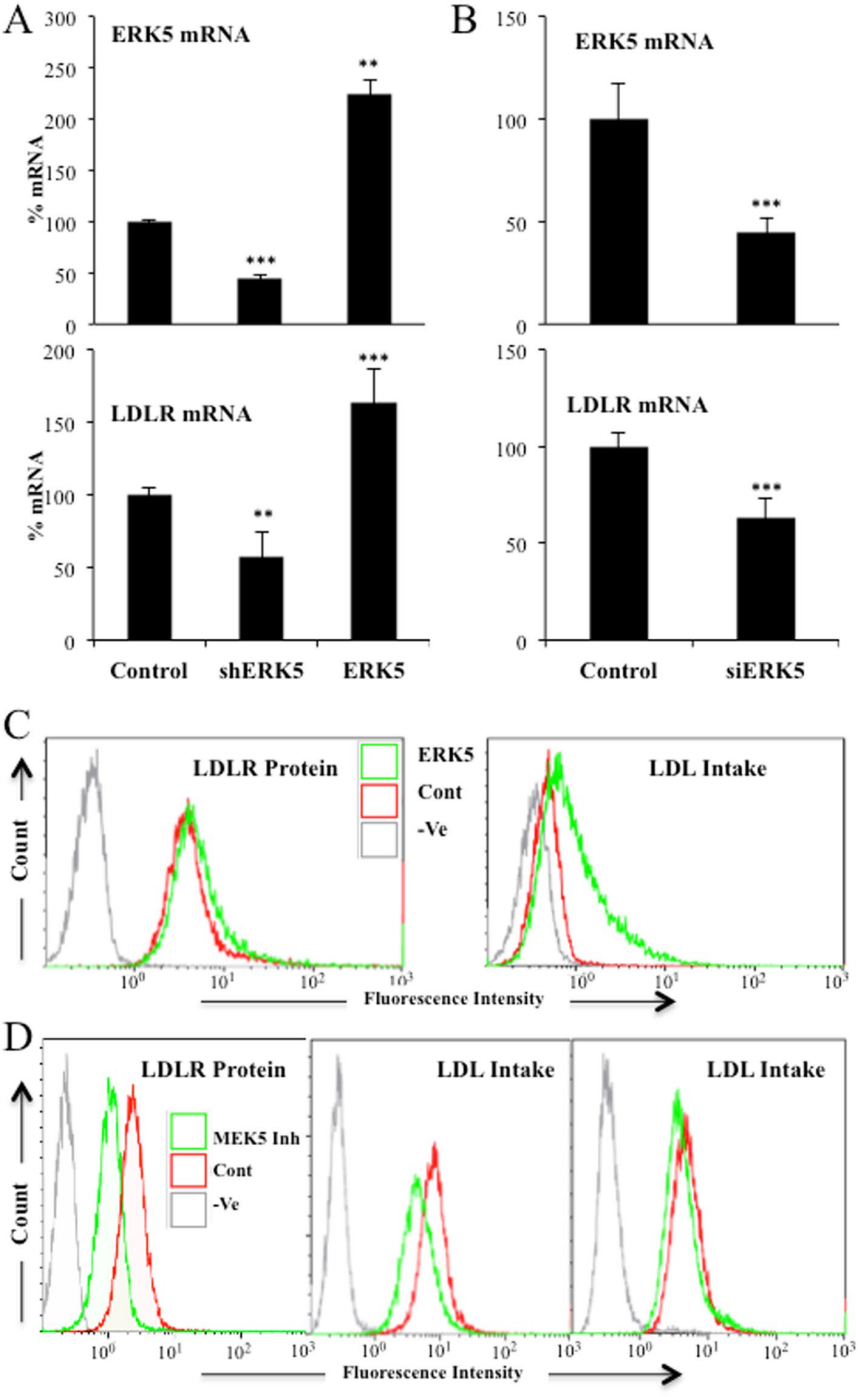
ERK5 controled LDLR expression and LDL uptake. (**A**) 10^7^ Jurkat-TAg cells were transfected with 5 µg of the empty pSUPER Neo vector (control) or with this vector containing a small hairpin RNA for ERK5 (shERK5) or with a pcDNA vector expressing ERK5 (ERK5). Forty-eight hours later mRNA expression of the whole population was analyzed by qPCR and represented as the % of mRNA compared to cells transfected with the empty vector. (**B**) Primary hepatocytes were transfected with control siRNA (control) or with siRNA against ERK5 (siERK5). 96 h later mRNA was collected and ERK5 and *LDLR* mRNA expression was analyzed by qPCR. (**C**) Jurkat cells were transfected with ERK5 as described in (**A**) and LDLR plasma membrane protein (left) and LDL intake (right) were analyzed by FACs. D) Jurkat (left and center) and OCI-AML3 (right) cells were treated with 5 μM of the MEK5 inhibitor BIX02189 for 24 h and LDLR protein (left) or LDL intake (center and right) were analyzed by FACs. Bar graphs represent means ± SD; *p < 0.05, **p < 0.01, ***p < 0.005 student t-test compared to empty vector transfected cells (control).

We further investigated the underlying mechanism of DCA-induced LDLR expression and the role of the ERK5/MEF2 pathway, which is activated by DCA^8, 10, 23, 24^. To this end, we targeted ERK5 utilizing a small hairpin RNA (shERK5). Reducing ERK5 expression resulted in decreased *LDLR* mRNA levels in non-treated hematopoietic cells (Fig. 5A). We could not investigate DCA effects on cells expressing shERK5 because ERK5 is essential to perform OXPHOS and hence DCA is highly toxic in cells with reduced ERK5 levels^8, 10, 23, 24^. Conversely, overexpression of ERK5 increased *LDLR* mRNA levels (Fig. 5A). As shown in Supplemental Fig. 4A, transfection with shERK5 or ERK5 expressing vectors efficiently decreased and increased ERK5 protein levels respectively. Decreasing ERK5 levels with small interference RNA for ERK5 (siERK5) also impaired LDLR expression in primary hepatocytes (Fig. 5B) or in HuH7 hepatic cells (Supplemental Fig. 5A), in which we observed a reduction of 40% on ERK5 protein levels (Supplemental Fig. 4B). Overexpression of ERK5 in Jurkat cells augmented LDLR protein and enhanced LDL uptake (Fig. 5C). The MAPKK MEK5 activates ERK5 in different physiological contexts^35^. Thus, we next used the MEK5 inhibitor BIX02189, which inhibits its catalytic function, and showed that it decreased LDLR protein and LDL uptake in Jurkat and OCI-AML3 cells (Fig. 5D). We validated these findings in primary tumor cells derived from a BCL patient (Supplemental Fig. 6). Treatment of these cells with an extremely selective ERK5 inhibitor, XMD8–92, resulted in the reduction of LDLR protein (Supplemental Fig. 6). Taken together, these results indicate that ERK5 is essential for LDLR expression and function in multiple cell lines.

### AMPK does not mediate DCA-induced LDLR expression

**Figure 6:**
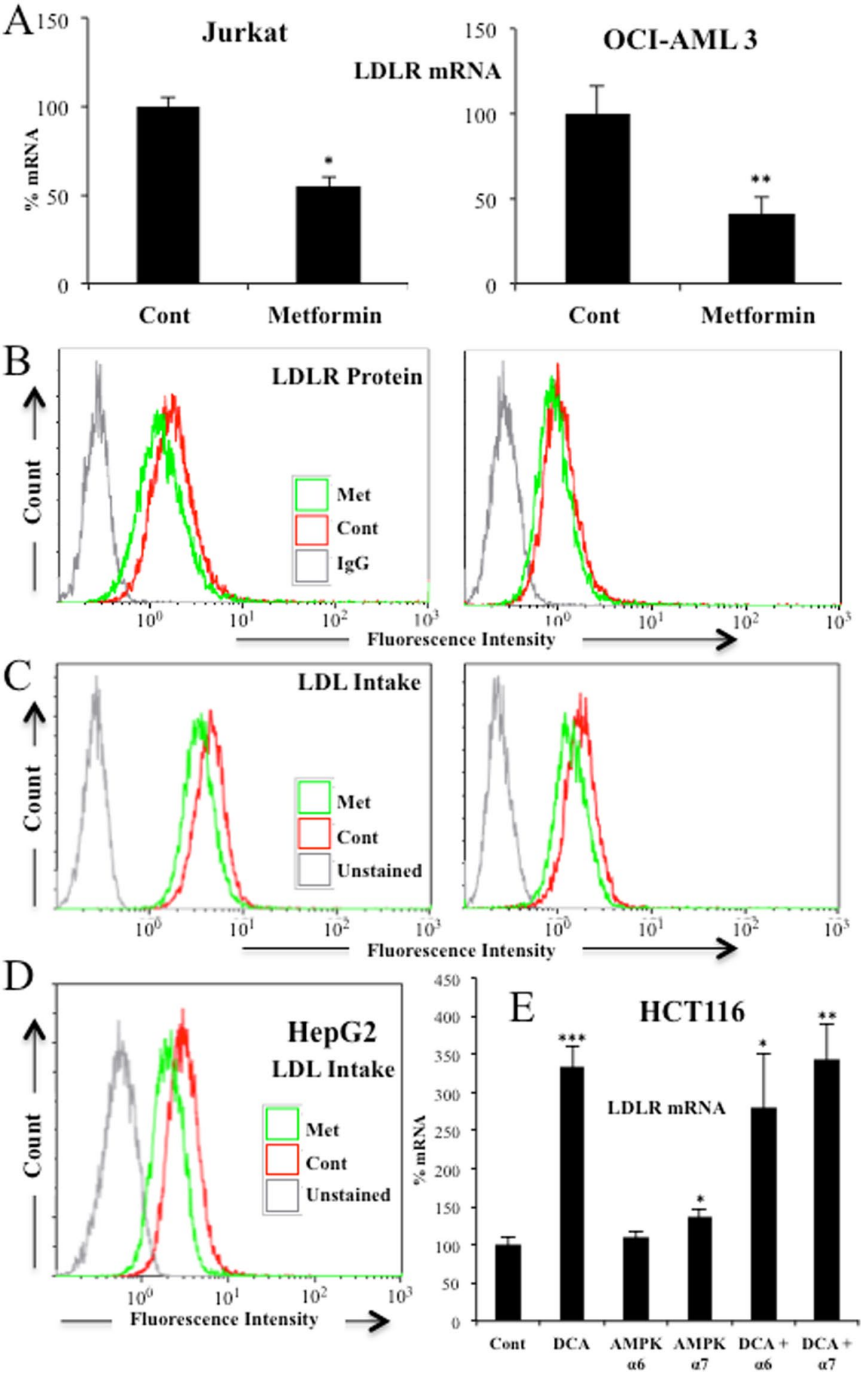
AMPK did not regulate DCA-induced LDLR expression and LDL uptake. Two different hematopoietic cell lines were treated with 5 mM metformin for 24 h and *LDLR* mRNA (**A**), protein (**B**) and LDL uptake (**C**) were analyzed. (**D**) HepG2-C3A cells were treated as in (**A**) and LDL uptake was measured. (**E**) HCT116 cells were transfected with 2 small interference RNA (siRNA) for AMPKα or with control siRNA and treated with 20 mM DCA for 6 h before mRNA analysis.

DCA induces AMPK activation, the main cellular metabolic sensor^36^. AMPK, by blocking *de novo* cholesterol production^16, 17^, could mediate LDLR expression as it has been observed with berberine or its analogs^18, 19^. The effect of berberine on LDLR expression was inhibited by PD98059^18^ and U0126^20, 21^, In fact, a PD98059^18^ and U0126^20, 21^ sensible pathway mediated berberine effect. Although these MAPKK inhibitors were initially described as specific MEK1 inhibitors, they also inhibit the ERK5 upstream kinase MEK5^37^. This indicates that DCA may have a similar mechanism of action than berberine. To test this hypothesis, we used metformin, which stimulates AMPK in Jurkat and OCI-AML cells^36^. Surprisingly, metformin did not increase, but rather decreased, *LDLR* mRNA levels (Fig. 6A), protein levels (Fig. 6B) and LDL intake (Fig. 6C) in two hematopoietic cell lines. Metformin also decreased LDL uptake in HEPG2-C3A cells (Fig. 6D). Moreover, blocking expression of the catalytic subunit of AMPK, AMPKα, with two different siRNA that effectively decrease AMPKα levels^38^, did not statistically decrease *LDLR* mRNA. In summary, these data indicated that the AMPK pathway did not modulate DCA-induced LDLR expression and suggested that we uncovered a totally new pathway that controlled LDLR expression.

### LDLR expression requires MEF2

**Figure 7:**
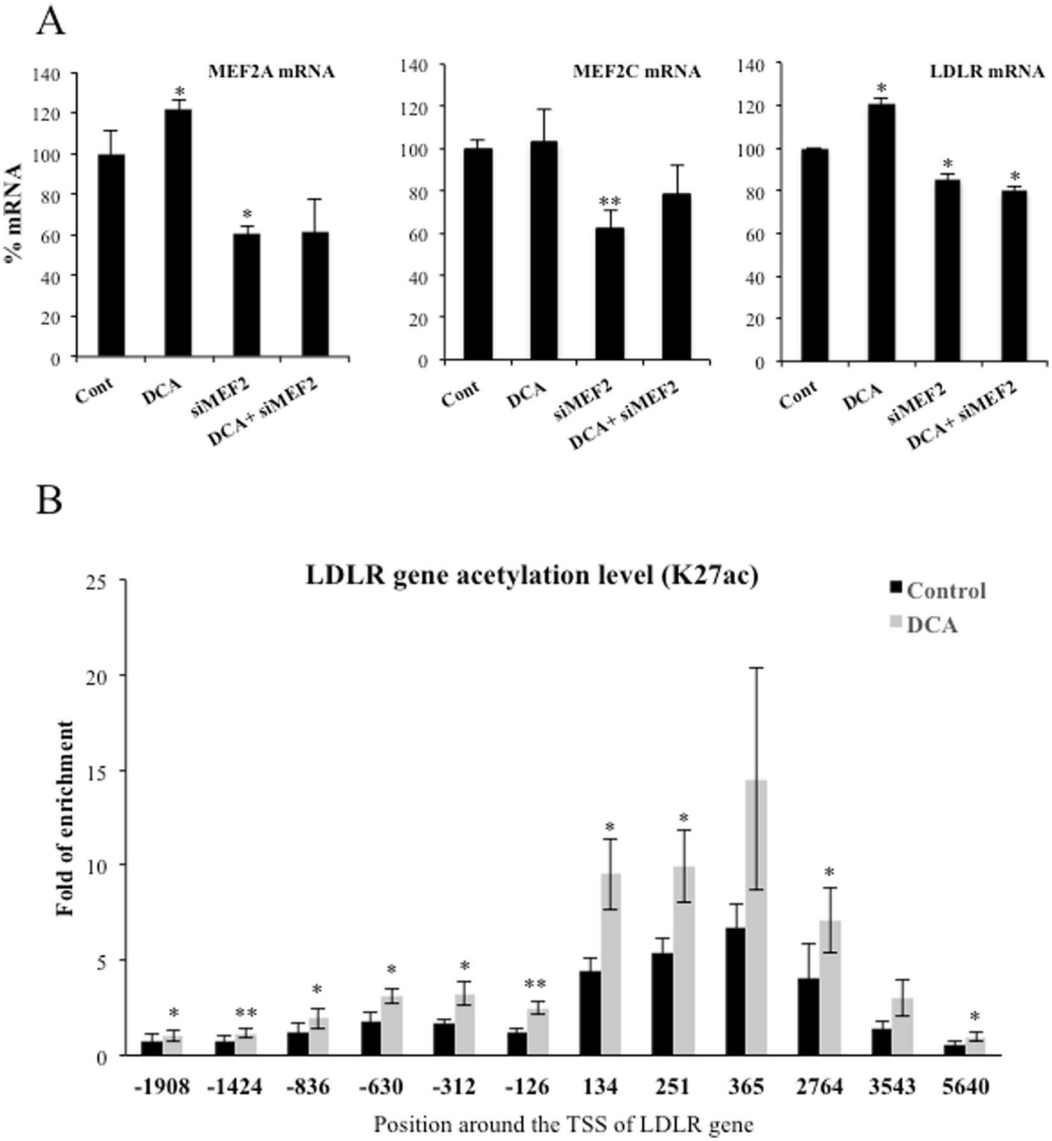
DCA required the transcription factor MEF2 to target LDLR promoter. (**A**) OCI-AML3 cells were transfected with 40 nM siRNA control or with 20 nM siRNA for each MEF2A and MEF2C (siMEF2). Twenty-four hours later cells were incubated for 24 h with 10 mM DCA. mRNA expression was analyzed by qPCR and represented as the % of mRNA compared to cells transfected with the empty vector. (**B**) OCI-AML3 cells were incubated for 72 h with 10 mM DCA. Cells were prepared for ChIP analysis using an antibody against H3 acetylation on lysine 27. Acetylation was revealed at different points of the LDLR promoter by using specific oligonucleotides. Bar graphs represent means ± SD; *p < 0.05, **p < 0.01, ***p < 0.005 student t-test compared to empty vector transfected cells (control).

ERK5 mediates part of its metabolic functions through the MEF2 family of transcription factors^8, 10, 23, 24^. Interestingly, *LDLR* promoter contains predicted binding sites for MEF2A and C that have been validated in several cell lines (http://genome.ucsc.edu/). Therefore, we performed a knockdown of MEF2A and C in OCI-AML3 cells by siRNA. These siRNA halved mRNA expression of both transcription factors (Fig. 7A) and reduced between 25 and 50% protein levels (Supplemental Fig. 4C). The level of knockdown was sufficient to significantly decrease *LDLR* mRNA levels (Fig. 7A). Finally, to further investigate whether DCA activated *LDLR* promoter, we checked the Histone H3 lysine 27 acetylation (H3K27Ac), a modification associated with active gene expression^39^. We observed that H3K27Ac increased after DCA treatment indicating an increase of *LDLR* gene transcription (Fig. 7B).

### The ERK5/MEF2 pathway also controls expression of the LDL receptor-adapter protein 1 (LDLRAP1)

**Figure 8:**
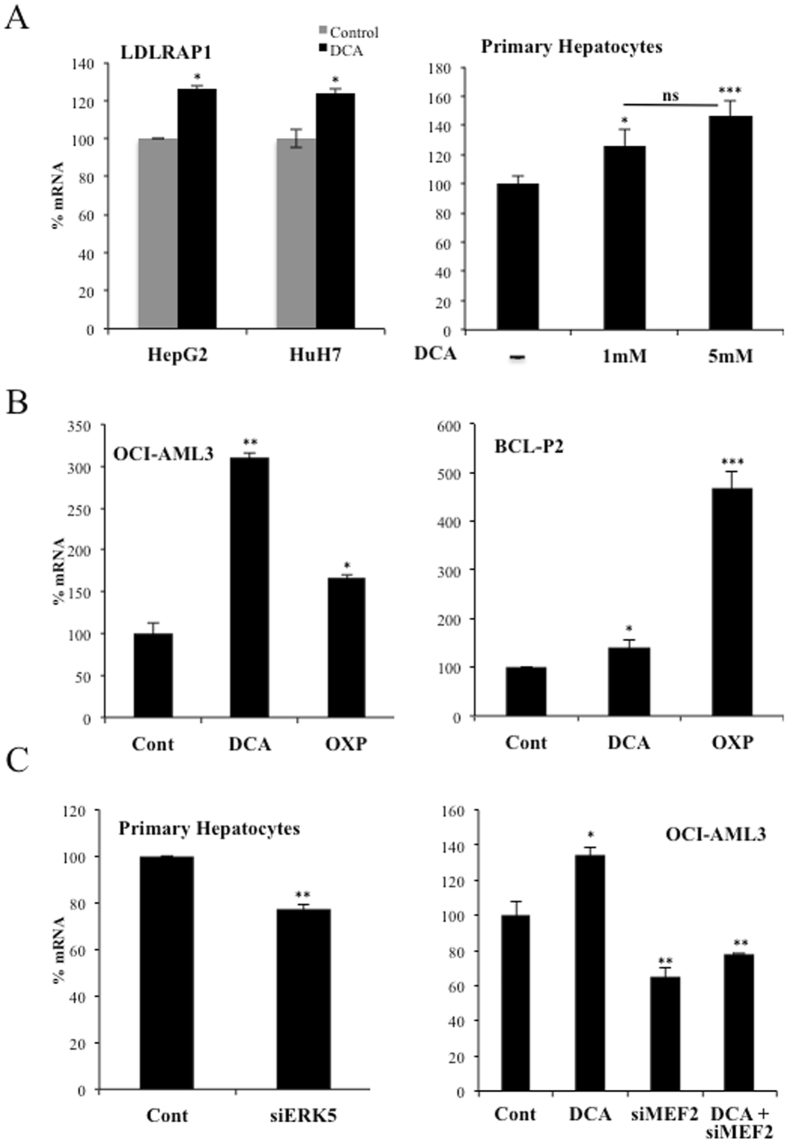
DCA induced LDLRAP1 expression. (**A**) Two hepatic cell lines or primary hepatocytes were treated with DCA as in Figs 1 and 2 and *LDLRAP1* mRNA was analyzed. (**B**) OCI-AML3 cells (left) and primary cells from a BCL patient (BCL-P2; right) were treated with 5 mM DCA or grown in OXPHOS medium for 2 weeks and *LDLRAP1* mRNA was measured. (**C**) Primary hepatocytes were transfected as in Fig. 5 and expression of *LDLRAP1* was analyzed by q-PCR. The bar graphs represent means ± SD of 3 independent experiments performed in triplicate; *p < 0.05, **p < 0.01, ***p < 0.005 student t-test or one-way ANOVA with post-hoc Tukey test.

The LDL receptor-adapter protein 1 (LDLRAP1) is a cytosolic protein that interacts with the cytoplasmic tail of LDLR^40^. *LDLRAP1* promoter contains MEF2 binding sites (http://genome.ucsc.edu/), suggesting that the same ERK5/MEF2 pathway could regulate this gene. Consequently with this hypothesis, DCA enhanced *LDLRAP1* expression in hepatic cell lines and primary hepatocytes (Fig. 8A). OCI-AML3 and primary tumor B cells also increased *LDLRAP1* mRNA after DCA treatment or after incubation in OXPHOS medium (Fig. 8B). In non-stimulated cells, siERK5 reduced *LDLRAP1* mRNA in primary hepatocytes, Huh7 cells and primary tumor cells (Fig. 8C (left), Supplementary Fig. 5C). Similarly, OCI-AML3 cells transfected with siMEF2 repressed the expression of *LDLRAP1* mRNA (Fig. 8C (right). In summary, cells performing OXPHOS increased the expression of an additional protein involved in LDLR activity.

## Discussion

Carbohydrate and lipid metabolism are intrinsically bound and their dysfunction play a major role in cardiovascular disease. Diabetes is typically associated to dyslipidemia, but *vice versa*, lipid changes also disturb glucose metabolism^41^. DCA, by stimulating PDH activity, decreases glucose catabolism and stimulates OXPHOS^6, 7^. To fuel it, cells could rely on fatty acid oxidation (FAO), suggesting that DCA could increase lipid catabolism. LDL particles transport cholesterol and triglycerides; hence an increase in LDLR should allow cells to increase fat availability. We propose that the avidity for fatty acids induces DCA-treated cells to increase LDLR expression and, subsequently, cholesterol uptake. This is supported by our observations showing that OXPHOS, which mediates FAO, reproduces DCA effects on LDLR expression *in vitro* (Fig. 1 and Supplemental Figs 2 and 3). The mechanism requires ERK5, which directs the choice of catabolic substrates^8, 10, 23–26^ and, hence, is a good candidate to control increase on fat avidity. Inhibiting ERK5 function by pharmacological or genetic means decrease LDL intake (Fig. 5). This reduces the consumption of exogenous cholesterol but should also affect the consumption of fatty acids. In contrast, *in vivo* ERK5 stimulation, e.x; by DCA treatment, induces *LDLR* mRNA (Fig. 4) and would induce triglyceride oxidation as previously observed^5, 14^.

The ERK5 target MEF2 also regulates this pathway, likely because *LDLR* promoter contains MEF2 binding sites (see Introduction). ERK5 induces activation of several members of the MEF2 family of transcription factors by several mechanisms. It induces direct phosphorylation at different serines and threonines on MEF2A, C and D^27, 28^. It can also activate MEF2A and D by direct interaction because ERK5 serves as a MEF2 coactivator through its signal-dependent direct association with the MEF2 MADS domain. Although, at least MEF2A-dependent transcription requires ERK5 kinase activity^29, 30^. MEF2 mediates several ERK5 effects on metabolism^10^. However, the exact mechanism via which DCA stimulates LDLR expression could be multifactorial, although it is a general phenomenon that we have confirmed in several hematopoietic, colon and hepatic cells (Fig. 1), including primary human hepatocytes (Fig. 2), which are the main regulators of cholesterol levels^2, 3^.

We cannot exclude that other mechanisms play a role in DCA-induced LDLR expression. For example, DCA inhibits HMG CoA reductase activity in liver and leukocytes^6^. This could lead to an even higher demand on exogenous cholesterol and subsequently to an increase in LDLR levels. In addition, we have also recently shown that the MAPK ERK5 induces Sirt1 expression^24^ that also stabilizes LDLR protein^42^. Therefore, ERK5 could target LDLR function in multiple ways and some of them independently of MEF2 family. In contrast, we have mainly excluded that ROS levels or AMPK activation play a major role in this process (Figs 3 and 6).

Other drugs could modulate the ERK5/MEF2 pathway to regulate the expression of LDLR. Berberine induces LDLR expression by a MAPK pathway sensitive to PD98059^18^ and U0126^20, 21^, two inhibitors that block the ERK5 pathway^37^. Then, ERK5 could also partly mediate berberine effects, although independently of AMPK (Fig. 6).

DCA decreases cholesterol plasma levels in several animal models and humans^5, 13–15^. In this study, we have mainly used high (10 mM) DCA concentrations for acute responses and “physiological” concentrations (1 to 5 mM) for chronic treatments. These latter values are in the range of those used in DCA-treated patients^11, 12^. Michelakis *et al*. gave 50 mg/Kg/day of DCA to patients^12^. On average, this amount of DCA should give a blood concentration of 4.6 mM, i.e. by considering 70 Kg/patient and a total of 5 L of blood. However, DCA plasma concentration was around 0.4 mM^11, 12^ and the ultimate destination of the DCA that was not in blood was unknown. Perhaps the liver processes DCA very fast as suggested by our results using primary hepatocytes (Fig. 2). There was an attempt to use DCA for treating hypercholesterolemia using similar DCA doses to ours *in vivo* ^15^. DCA reduced circulating cholesterol levels in two patients through a mechanism involving a reduction in LDL cholesterol^15^, although both patients initially showed low LDLR surface activity. However, DCA was halted due to its neuropathological effects^15^ and this precluded its use to treat high cholesterol. These pathological effects have been observed in other clinical contexts, e.g. lactic acidosis and stroke-like episodes (MELAS)^43^. At the beginning of their DCA treatment, patients quickly eliminate DCA^11, 12^. In this manuscript, we have observed that in primary hepatocytes fresh DCA should be daily added to media to keep physiological effects (Fig. 2). As expected, hepatocytes probably metabolize DCA faster than other cell types^6^. After several weeks of treatment, DCA plasma concentrations reach a plateau around 0.4 mM^11, 12^, and during this phase neuropathology appears^11^. From a pharmaceutical point of view, it would be desirable to use drugs with the LDLR-stimulating effect of DCA, but without its neuropathological effects. The uncovering of ERK5/MEF2 pathway as a regulator of LDLR expression opens an interesting pharmacological possibility.

## Methods

### Ethical statement

Experimental procedures were conducted according to the European guidelines for animal welfare (2010/63/EU). Protocols were approved by the Animal Care and Use Committee “Languedoc-Roussillon” (approval number: CEEA-LR-12163). The use of human specimens for scientific purposes was approved by the French National Ethics Committee. All methods were carried out in accordance with the approved guidelines and regulations of this committee. Written informed consent was obtained from each patient prior to surgery.

### *In vivo* mouse experiments

*In vivo* experiments were carried out using 6 to 8 weeks/old male NSG mice. Mice were bred and housed in pathogen-free conditions in the animal facility of the European Institute of Oncology–Italian Foundation for Cancer Research (FIRC), Institute of Molecular Oncology (Milan, Italy). For engraftment of human cells, 1 million AML cells were injected intravenously (i.v.) through the lateral tail vein in non-irradiated mice. NSG mice with established human AML tumors (day 80 post-graft) were treated with DCA (50 mg/kg, 1 dose/day by gavage, starting at day 1 for 16 consecutive days). Human tumor AML cells gather in mouse spleen and bone marrow, hence we isolated mRNA from these organs. We used human-specific primers to visualize expression of human *LDLR* mRNA. In a different experiment B6 wt mice were treated with a daily single dose of DCA (50 mg/kg/day) intraperitoneally and mouse *LDLR* mRNA was analyzed in spleen and liver at different time points.

### Cell lines and culture conditions

The leukemic human cell lines T Jurkat TAg and OCI-AML3 were grown in RPMI 1640–Glutamax (GIBCO) supplemented with 5% (Jurkat) or 10% (OCI-AML3) FBS^10, 24^. Primary cells from a lymphoma B cell patient (BCL-P2) were grown in the same medium with 10% FBS. In certain experiments cells were grown in RPMI 1640 without glucose (GIBCO 11879) with the addition of 2 mM glutamine and 10 mM galactose (OXPHOS medium). The Jurkat TAg cells carry the SV40 large T Ag to facilitate cell transfection. HepG2-C3A and HuH7 cells were grown in MEM and DMEM respectively supplemented with FBS, sodium pyruvate, glutamine, penicillin and streptomycin. The HCT116 human colon cancer cells were cultured in low glucose (5 mM) DMEM medium supplemented with 10% FBS. Cellular confluence during experiments was between 80–85%.

### Human liver samples and preparation of PHHs cultures

Liver samples were obtained from liver resections performed in adult patients for medical reasons. Human hepatocytes isolation and culture were performed as described previously^44^. Briefly, after liver perfusion, hepatocytes were counted and cell viability was assessed by trypan blue exclusion test. A suspension of 1 × 10^6^ cells/mL per well was added in 12-well plates pre-coated with type I collagen (Beckton Dickinson) and cells were allowed to attach for 12 h. Then, the supernatant containing dead cells and debris was carefully removed and replaced with 1 mL of serum-free long-term culture medium (Lanford medium, LNF). The number of confluent attached cells was estimated at ~1.5 × 10^5^ cells/cm^2^.

### Reagents and antibodies

DCA was from Santa Cruz Technologies. Galactose and glutamine were from GIBCO. Human anti-LDLR-PE and IgG were from BD Biosciences and 7AAD from Beckman. The MEK5 inhibitor BIX02189 and the ERK5 inhibitor XMD8–92 were from Selleck. RIPA buffer to prepare protein extracts was from Euromedex. The complete protease inhibitor cocktail (Complete EDTA-free) and the phosphatase inhibitor cocktail (PhosSTOP) were from Roche. ERK5 and MEF2A/C antibodies were from Cell Signaling Technology and Abcam respectively. The antibody against β-Actin and HRP-labeled secondary antibodies were from Sigma.

### Transient transfection

Jurkat cells in logarithmic growth phase were transfected with the indicated amounts of plasmid by electroporation^45, 46^. In each experiment, cells were transfected with the same total amount of DNA by supplementing with empty vector. Cells were incubated for 10 min at RT with the DNA mix and electroporated using the Gene Pulser Xcell™ Electroporation system (Bio-Rad) at 260 mV, 960 mF in 400 µl of RPMI 1640. Expression of the different proteins was confirmed by western blot. The transfection efficiency in Jurkat TAg cells is between 60 and 80%. OC-AML-3 cells were transfected using Amaxa ^TM^ D-Nucleofector ^TM^ Lonza Kit according to manufactured protocol. In HuH7 and HCT116 cells, transfection of 30–50 nM siRNAs was carried out using Lipofectamine RNAiMAX (Invitrogen) in Opti-MEM (Invitrogen), according to the manufacturer’s instructions. Primary hepatocytes were transfected twice at days 1 and 3 post-seeding. Cells were harvested 48 to 96 h post-transfection.

### Plasmids

The expression vectors for ERK5, the pSUPER expression vector for GFP alone or GFP plus shERK5 and the pSiren-retroQ-puro (BD Biosciences) retroviral vectors for shERK5 and control have been previously described^45^. Control, MEF2A and C and ERK5 siRNA were ON-TARGETplus SMARTpools (mixture of 4 siRNA) were from Dharmacon.

### Counting and determination of cell viability

Cell number, viability and cell death was analyzed with the Muse Cell Analyzer (Millipore) by incubating cells with Muse Count & Viability and Annexin V and Dead Cell kits respectively, following manufacturer’s instructions.

### ROS measurement

Cells lines were plated at 300,000 cells/ml and treated with DCA for the indicated times, harvested and counted to perform further analysis. To evaluate ROS levels, we labeled cells with CellROX® Deep Red Reagent or with CH-H2DCFDA (Life Technologies) for 30 minutes and analyzed them by FACs following manufacturer’s instructions.

### RT-PCR and DNA sequencing

Total RNA was extracted using NucleoSpin RNA isolation columns (Macherey-Nagel), reverse transcription was carried out using iScript™ cDNA Synthesis Kit (Biorad). Quantitative PCR was performed with KAPA SYBR Green qPCR SuperMix (Cliniscience) and a CFX Connect™ Real-Time qPCR machine (Biorad) with LDLR, LDLRAP1, ERK5 and actin primers (Supplemented Fig. 7). All samples were normalized to β-actin mRNA levels. Results are expressed relative to control values arbitrarily set at 100.

### Immunoblotting

Protein analysis by immunoblotting was performed essentially as previously described^45^. Briefly, samples were collected, washed out with PBS and lysed with RIPA buffer. Protein concentration was determined by BCA assay (Pierce) before electrophoresis in 4–15% TGX gels (BioRad) and equal amount of protein was loaded in each well. Protein transfer was performed in TransTurbo system (BioRad) in PVDF membranes. After blocking for 1 h with 5% non-fat milk, membranes were incubated overnight at 4 °C in agitation with primary antibodies, washed three times with PBS-Tween 0,1% and incubated with the appropriate HRP-labeled secondary antibody for 1 h. Membranes were washed out three times with PBS-Tween 0,1% and developed with Substrat HRP Immobilon Western (Millipore). Band quantification was performed using the “ImageLab” software from BioRad and represented as the ratio between the protein of interest and a control protein i.e. actin. The value of 1 is arbitrarily given to control cells. One blot representative of several experiments is shown.

### LDL Intake

After treatment cells were incubated with BODIPY FL LDL (Invitrogen) in PBS with 2% FBS and incubated at 37 °C for 30 min. Cells were then washed and suspended in 200–250 μl PBS 2% FBS and analyzed using a Gallios flow cytometer (Beckman) and the Kaluza software.

### Flow Cytometry

Briefly, 1 × 10^6^ cells were stained with antibody in PBS with 2% FBS and incubated at 37 °C for 30 min. Cells were then washed and suspended in 200–250 μl PBS 2% FBS and staining was analyzed using a Gallios flow cytometer (Beckman) and the Kaluza software.

### ChIP analysis

OCI-AML3 cells were treated with 10 mM DCA for 72 h. Ten million cells were centrifuged (5 min; 1200 rpm) and the pellet was washed two times in 1X phosphate-buffered saline (PBS) at room temperature and suspended in 10 mL of 1X PBS. Cells were fixed in 1% paraformaldehyde (Electron Microscopy Sciences) at room temperature for 5 min. Fixation wa lysed in 1 ml of cell lysis buffer (5 mM PIPES, 85 mM KCl, 0.5% NP40, Na Butyrate 10 mM + 2X protease inhibitor cocktail (Halt™ Protease Inhibitor Cocktail, EDTA-Free (100X), Thermofischer)) at 0 °C for 10 min. Nuclei were recovered by centrifugation (10 min, 5000 rpm) at 4 °C and lysed in 250 µl nuclei lysis buffer (50 mM Tris-HCl pH 7.5, 1% SDS, 10 mM EDTA, Na Butyrate 10 mM + Halt™ Protease Inhibitor Cocktail (3X)) at 4 °C for at least 2 hours. 250 µl of each sample were then sonicated 2 times for 5 min (30 s on/off) at 4◦C using a Bioruptor (Diagenode). After sonication, absorbances at 280 nm (A280) of 1/100 diluted samples were measured and A280nm and was adjusted to 0.133 with nuclei lysis buffer. One hundred microliter were used for ChIP experiments in a final volume of 1 ml. Samples were incubated under gentle agitation at 4 °C overnight in the presence of 3 µg of either a specific antibody or a negative control. Antibodies (anti-K27Ac Ab4729 (Abcam) and negative control IgG (Diagenode)) were previously bound to DYNA Beads Protein G Novex (Life Technology) according to the supplier’s recommendations. Dynabeads-bound immunoprecipitates were sequentially washed once with a low salt buffer (50 mM Tris-HCl pH 7.5, 150 mM NaCl, 1% triton, 0.1% SDS, 1 mM EDTA, 1 mM Na Butyrate + Halt™ Protease Inhibitor Cocktail (1X)), a high-salt buffer (50 mM Tris-HCl pH 7.5, 500 mM NaCl, 1% triton, 0.1% SDS, 1 mM EDTA, 1 mM Na Butyrate) and a LiCl-containing buffer (20 mM Tris-HCl pH 7.5, 250 mM LiCl, 1% NP40, 1% Na deoxycholate, 1 mM EDTA, 1 mM Na Butyrate) and, then, twice with a TE buffer (10 mM Tris-HCl pH 7.5, 1 mM EDTA, Tween 20 0.02%). Samples were then eluted in 250 µL of elution buffer (100 mM NaHCO3, 1% SDS), and DNA-protein complexes were incubated at 65 °C for 5 hours to reverse crosslinks. Samples were then treated with 100 mg/ml proteinase K and 100 mg/ml RNAse A at 45 °C for 2 hours to digest proteins and contaminating RNA. DNA was purified with an extraction kit (NucleoSpin Gel and PCR clean-up, Macherey-Nagel) according to the manufacturer’s recommendations and qPCR analysis was performed using the Roche LightCycler 480 real-time PCR system. The data were normalized with inputs taken from samples before the immunoprecipitation and treated under the same conditions. The primers used to amplify various regions of LDLR gene promoter.

### Statistical analysis

The statistical analysis of the difference between means of paired samples was performed using the paired t test. Analysis of multiple comparisons with single control was performed using one-way ANOVA with post-hoc tukey test. The results are given as the confidence interval (*p < 0.05, **p < 0.01, ***p < 0.005). All the experiments described in the figures with a quantitative analysis have been performed at least three times in duplicate. Other experiments were performed three times with similar results.

### Data availability

All data are available upon request.

## Electronic supplementary material

Supplementary Information
