# Supplementary material for: The PDK1 Inhibitor Dichloroacetate Controls Cholesterol Homeostasis Through the ERK5/MEF2 Pathway
Source: Sci Rep. 2017 Sep 6;7:10654. doi: 10.1038/s41598-017-10339-5 (PMC5587676; doi:10.1038/s41598-017-10339-5)
Supplement: Supplementary file 1 — Supplementary Information [file 41598_2017_10339_MOESM1_ESM.pdf]

The PDK1 Inhibitor Dichloroacetate Controls Cholesterol Homeostasis Through the ERK5/MEF2 Pathway

Abrar Ul Haq Khan, Nerea Allende-Vega, Delphine Gitenay, Sabine Gerbal-Chaloin, Claire Gondeau, Dang-Nghiem Vo, Sana Belkahla, Stefania Orecchioni, Giovanna Talarico, Francesco Bertolini, Milica Bozic, Jose M. Valdivielso, Fabienne Bejjani, Isabelle Jariel, Isabel C. Lopez-Mejia, Lluis Fajas, Charles-Henri Lecellier, Javier Hernandez, Martine Daujat and Martin Villalba.

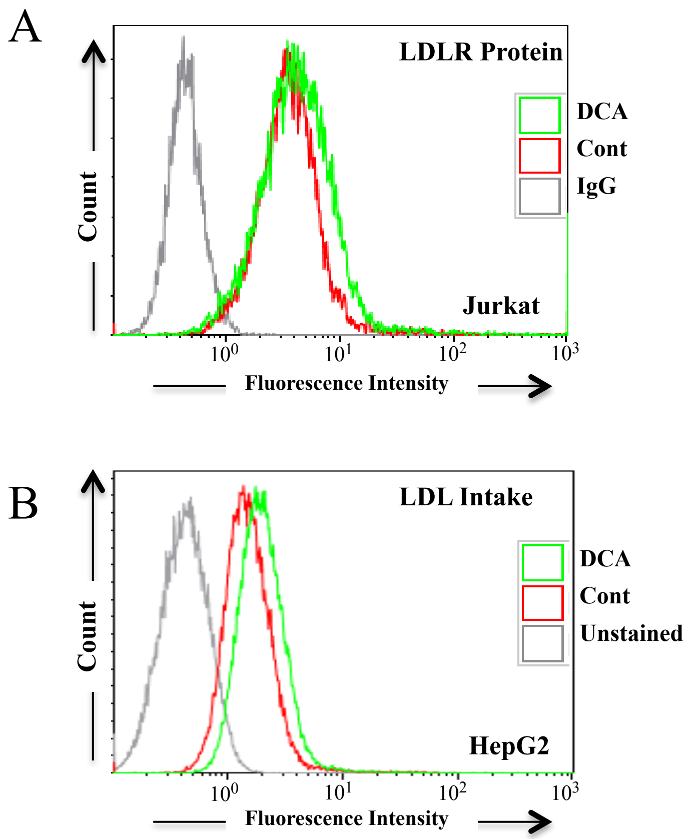

Supplemental Figure 1. A) Jurkat cells were treated with 10mM DCA for 24h and LDLR protein was analyzed by FACs.

B) HepG2 cells were treated with 10mM DCA for 72h and LDL intake was analyzed by FACs.

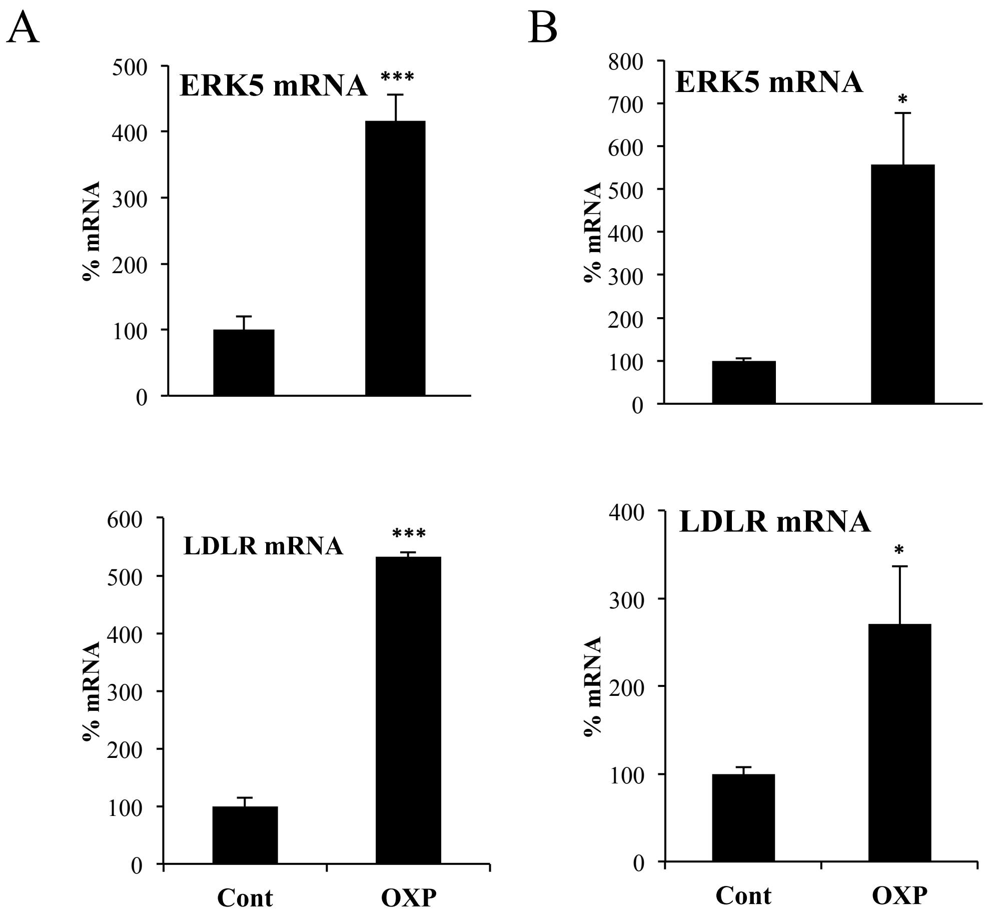

Supplemental Figure 2. (A) OCI- AML3 or (B)BCL-P2 cells were grown in OXPHOS medium for 2 weeks before mRNA analysis.

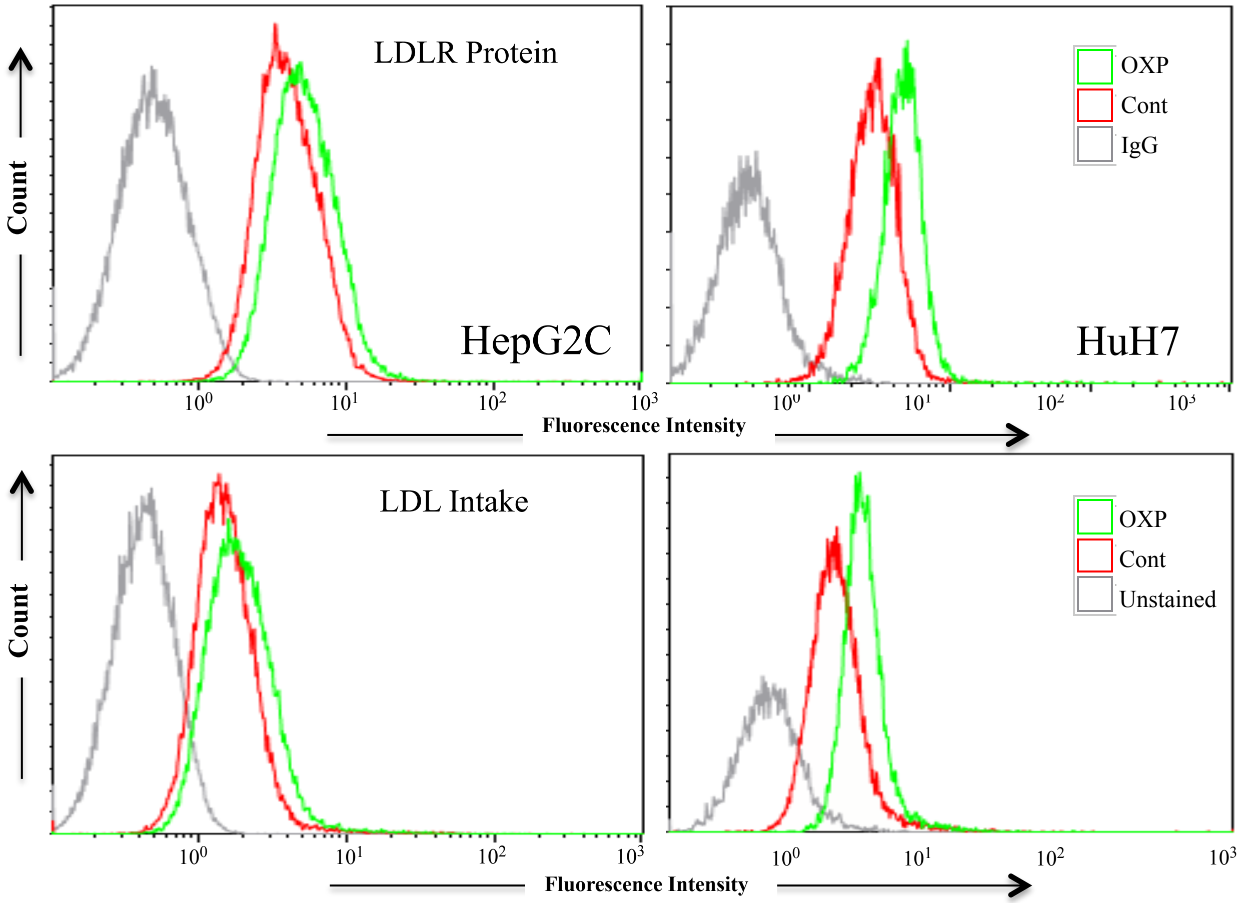

Supplemental Figure 3. HepG2 and HuH7 cells were grown in OXPHOS medium for 2 weeks before analyzing LDLR protein (upper panel) and LDL intake (lower panel) by FACs.

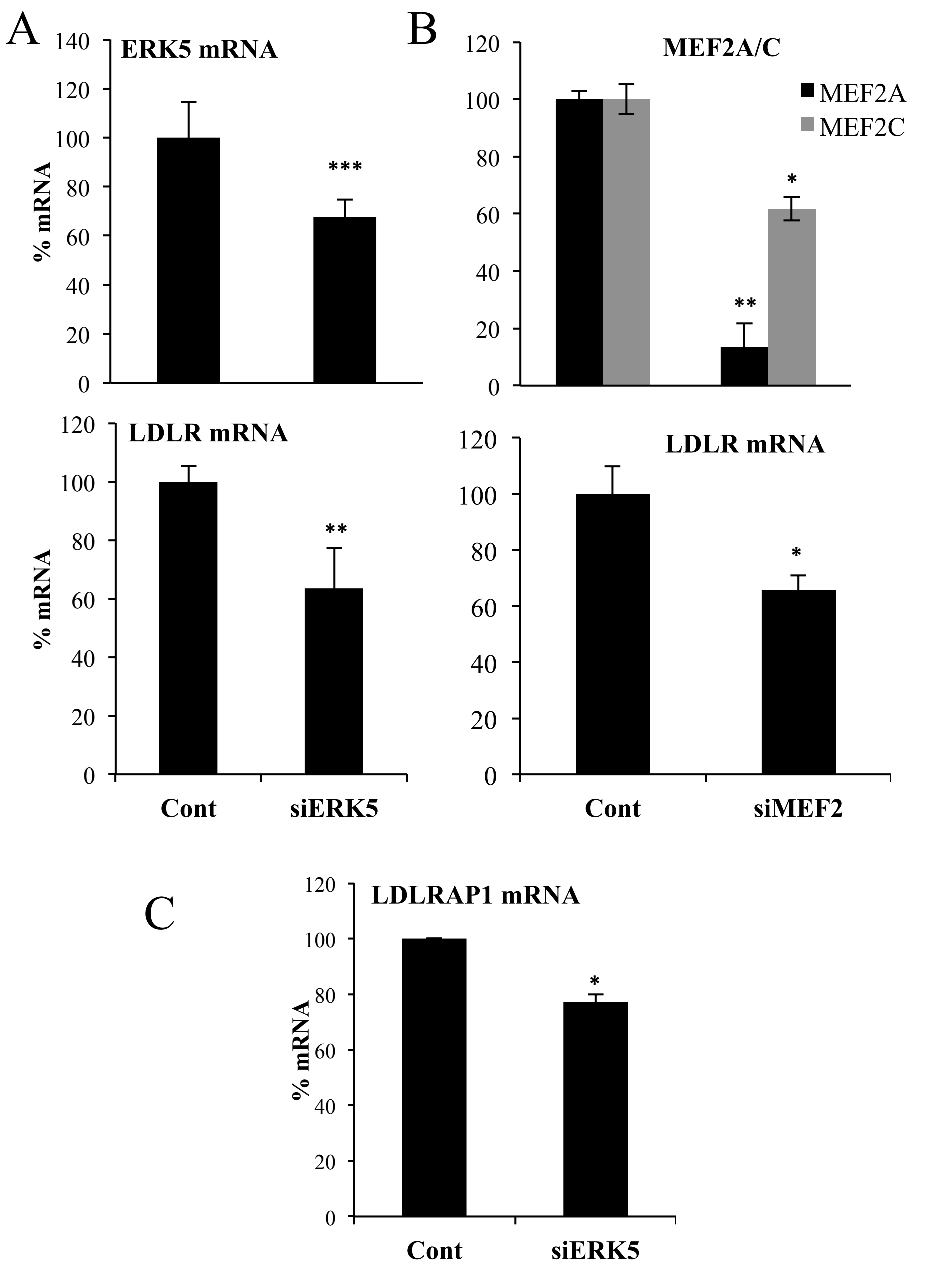

Supplemental Figure 4. A) Huh7 cells were transfected with control siRNA or with siRNA against ERK5 (siERK5). 36 h later mRNA was collected and ERK5 and LDLR mRNA expression was analyzed by qPCR

B) HepG2C cells were transfected with control siRNA or with siRNA against MEF2A and MEF2C (siMEF2A/C). 36 h later mRNA was collected and MEF2A/C and LDLR mRNA expression was analyzed by qPCR

C) LDLRAP1 mRNA was analyzed from Huh7 cells as in A

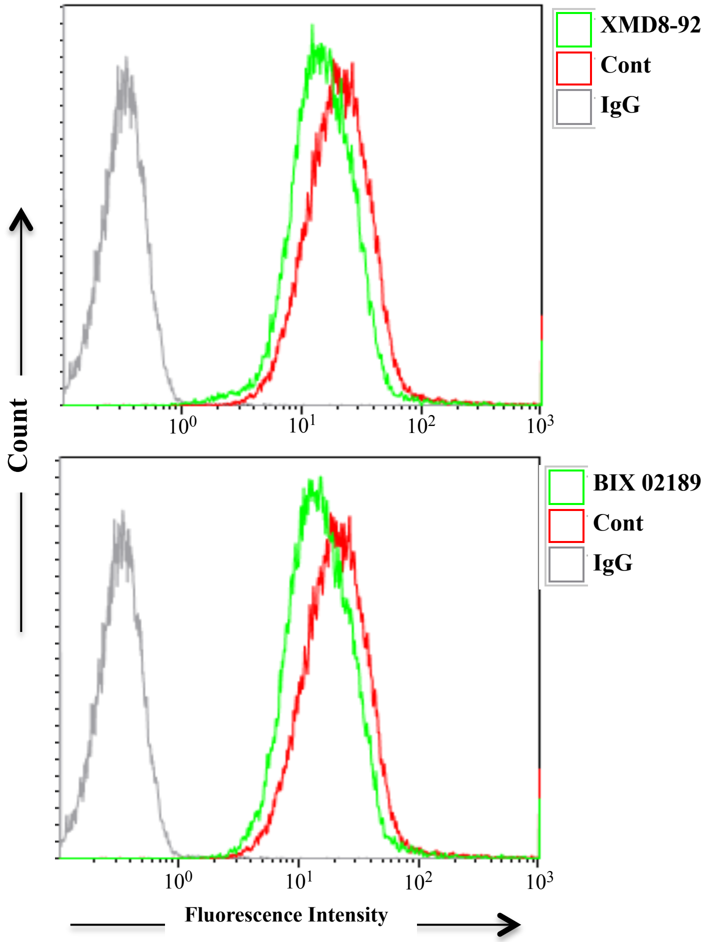

Supplemental Figure 5. Primary BCL-P2 cells were treated with 5 µM of the MEK5 inhibitor (BIX02189) or the ERK5 inhibitor(XMD8-92) for 48 h and LDLR protein was analyzed by Facs.

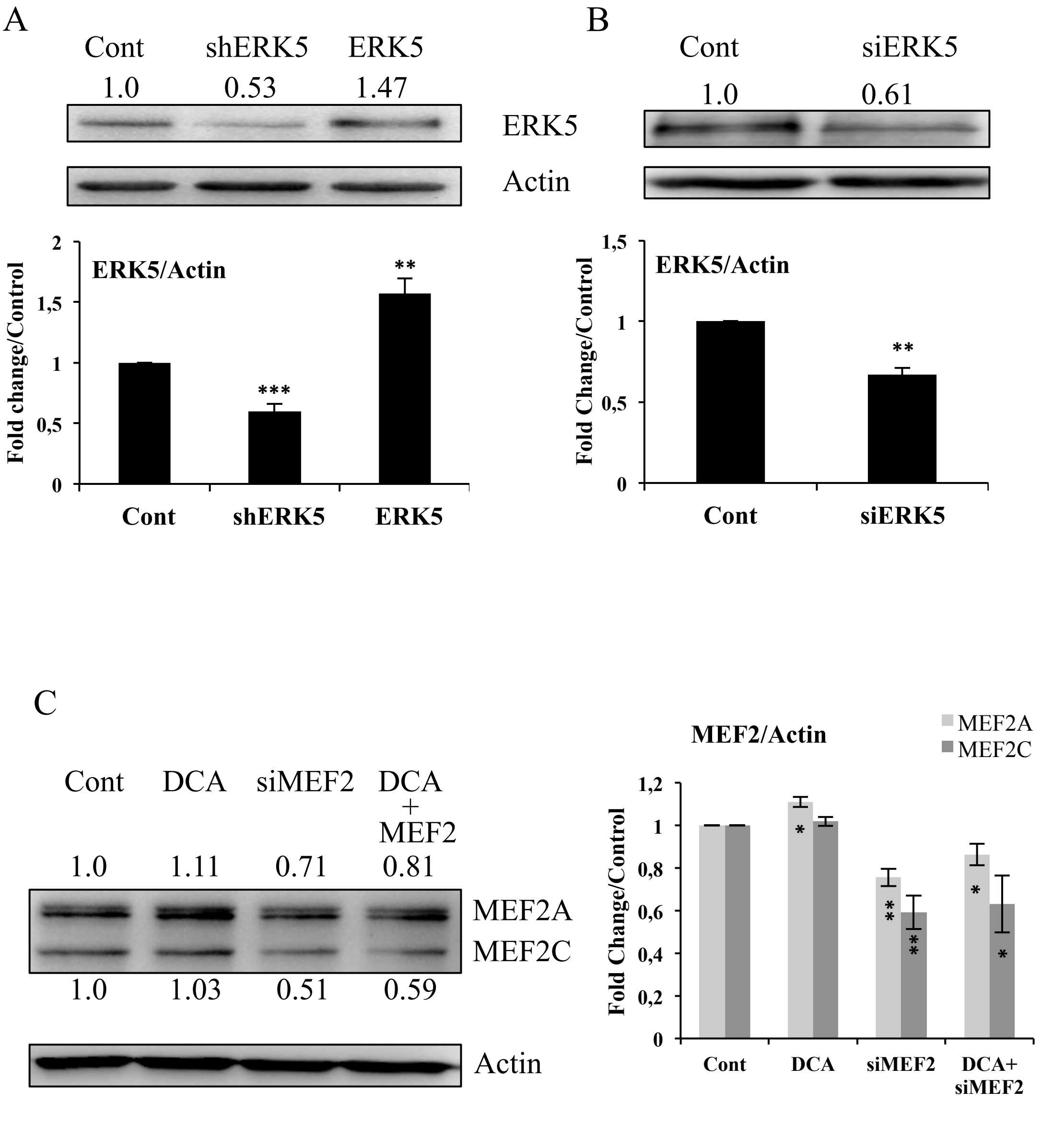

Supplemental Figure 6. A) Jurkat cells transfected as in Figure 5A and ERK5 protein was measured by WB.
B) HuH7 cells transfected as Supplemental Figure 4 A and ERK5 protein was measured by WB.
C) OCI-AML3 cell transfected with siMEF2 and MEF2 protein was measured by WB.

List of Primers:

LDLR (H):
Forward: *AGGGACCCAACAAGTTCAAG*
Reverse: *AAGAAGAGGTAGGCGATGGA*

ERK5 (H):
Forward: *CGCTACTTCCTGTACCAACTGC*
Reverse: *AGCCATACCAAAGTCACCAATC*

MEF2A (1) (H):
Forward: *CCAGCTGCTCCGGAGATACG*
Reverse: *GGAGTGAGTGCGGGAGACAA*

MEF2A (H):
Forward: *TGAGAGCCCTGATGCTGACG*
Reverse: *ACATCCCACCTGCATTGCCA*

MEF2C (1) (H):
Forward: *CATCCCAGTGTCCAGCCACA*
Reverse: *CCAGGTGAGACCAGCAGACC*

MEF2C (H):
Forward: *GCGTGCTGTGTGACTGTGAG*
Reverse: *TGTGGCTGGACACTGGGATG*

ACTIN (H):
Forward: *GAGGGAAATCGTGCGTGACA*
Reverse: *AATAGTGATGACCTGGCCGT*

GAPDH (H):
Forward: *CTGCACCACCAACTGCTTAG*
Reverse: *AGGTCCACCACTGACACGTT*

LDLR (M):
Forward: *GAATCTACTGGTCCGACCTGTC*
Reverse: *CTGTCCAGTAGATGTTGCGGTG*

ACTIN (M):
Forward: *GCGGACTGTTAGTGAGCTGCG*
Reverse: *TGTTTGCTCCAACCAACTGCTGTC*

GAPDH (M):
Forward: *CTACAGCAACAGGGTGGTGGAC*
Reverse: *GGGTGCAGCGAACTTTATTGATGG*

Supplemental Figure 7.
